# A telephone survey of factors affecting willingness to participate in health research surveys

**DOI:** 10.1186/s12889-015-2350-9

**Published:** 2015-10-05

**Authors:** DC Glass, HL Kelsall, C. Slegers, AB Forbes, B. Loff, D. Zion, L. Fritschi

**Affiliations:** Department of Epidemiology and Preventive Medicine, Monash University, The Alfred Centre, 99 Commercial Rd, Melbourne, 3004 VIC Australia; VU Human Research Ethics Committee, Office For Research, FP, Victoria University, PO Box 14428, Melbourne, VIC 8001 Australia; School of Public Health, Curtin University, Kent Street, Bentley, WA 6102 Australia

**Keywords:** Epidemiology, Population studies

## Abstract

**Background:**

In recent years, reduced participation has been encountered across all epidemiological study designs, both in terms of non-response as well as refusal. A low response rate may reduce the statistical power but, more importantly, results may not be generalizable to the wider community.

**Methods:**

In a telephone survey of 1413 randomly selected members of the Australian general population and of 690 participants sourced from previous studies, we examined factors affecting people’s stated willingness to participate in health research.

**Results:**

The majority of participants (61 %) expressed willingness to participate in health research in general but the percentage increased when provided with more specific information about the research. People were more willing if they have personal experience of the disease under study, and if the study was funded by government or charity rather than pharmaceutical companies.

Participants from previous studies, older people and women were the groups most willing to participate. Younger men preferred online surveys, older people a written questionnaire, and few participants in any age and sex groups preferred a telephone questionnaire.

**Conclusion:**

Despite a trend toward reduced participation rates, most participants expressed their willingness to participate in health research. However, when seeking participants, researchers should be concrete and specific about the nature of the research they want to carry out. The preferred method of recommended contact varies with the demographic characteristics.

## Background

In recent years, reduced participation has been encountered across all epidemiological study designs [1]. Fewer potential participants are contactable [2], and a reduced proportion of those contacted respond to invitations [3–5] sometimes because the potential responder did not receive or did not take notice of the invitation [6]. There is also less likelihood that those contacted will participate; the refusal rate for telephone surveys has gone from 4 % in the 1950s and 60s to 53 % in 1990 [2]. In consumer surveys, the decline in telephone response rates increased between 1996 and 2003 [7].

Nonresponses and failure to make contact with possible participants based on a predetermined number of contact attempts stated in the study protocol, are a problem in epidemiology because they may result in smaller sample sizes and reduced power. More importantly however, the results from low response rates may not be generalizable to the wider community because the survey respondents may differ in important ways from the general population and so are not representative [2, 3, 8–12].

In this study we sought to understand how willing people are to participate in health research and investigate the factors that affect their willingness to participate. To this end, we organised a telephone survey of a random sample of Australians to identify their willingness to take part in heath research.

We also undertook the telephone survey in a sample of participants from an existing cohort and a previous case–control study to identify the willingness of known study participants to take part in another health study and to identify differences in their views compared to those who had not previously participated in such a study eg on the role of Ethics Committees [13].

## Methods

### Participants

In order establish willingness of the general Australian population to participate in health surveys we chose a random sample of the Australian population. We also solicited views from individuals who had previously taken part in health surveys to identify whether this had led to a change in willingness to participate.

The study population had three groups:

1. The general population.
2. Previous participants from a case-control study
3. Previous participants from a cohort study

To obtain the general population sample, a sample of 7000 randomly generated working telephone numbers was obtained from a commercial list provider. Of these, a geographically representative sample of 4000 landline telephone numbers were drawn from the capital city and non-metropolitan area of each state and territory in proportion to their population. In addition, 3000 mobile telephone numbers were drawn to ensure that we had adequate representation of individuals with mobile telephones who may not have landlines. In order to randomise the choice of the individual who was surveyed within each household, the interviewer asked to speak to the household member who was aged over 18 years and who had the next birthday.

For the cohort, a random sample of 600 individuals who were under 80 years, living in Australia and for whom a telephone number was available was drawn from an existing prospective occupational cohort. [14] (Table 1). The cohort was a cancer and mortality study of approximately 18,000 workers in the Australian petroleum industry of whom 90 % were male [15]. Employees had been enrolled in the cohort by participating in face to face interviews in at least one of four surveys between 1980 and 2000 which took place during work time with help from the site medical services. Over 90 % of eligible employees participated in the cohort. Refusal to participate was uncommon. The major cause of non-participation was difficulty in locating individuals because of temporary absences such as sick leave or annual leave [15].

**Table 1:** Study population and participation rates by study sample groups

	Complete study population						
	General population sample				Case–control study	Cohort study	Total study population
	Landline			Mobile telephone			
	Letter sent	No letter	All landline	No letter	Letter sent	Letter sent	
Study population approached *n*	1806	2194	4000	3000	200	600	7800
Non-contact/phone number unusable^a^ *n*	391	1123	1514	2005	33	77	3629
Eligible population^b^ *n*	1415	1071	2486	995	167	523	4171
Refusals^c^	761	631	1372	540	14	122	2068
Completed interviews *n*	654	440	1094	455	153	401	2103
Participation rate %	46.2	41.1	44.0	45.7	91.6	76.7	50.4

^a^Includes no answer, engaged, answering machine, those under 18, those with language difficulty and too frail/old/ill

^b^Excludes non-contacts

^c^Includes those who actively declined or claimed to have already done the survey, or made but did not keep an appointment, or terminated during the survey, or said that they did not know the named person or who asked to be removed from the list

The case-control study of bowel cancer in Western Australia recruited 918 cases diagnosed between 2005 and 2007 and controls selected from the Western Australian electoral roll [16].(National Electoral roll registration is compulsory for everyone 18 years and over.) The patients in WABOHS were men and women aged between 40 and 79 [17]. Participants had been asked if they would be prepared to take part in other related research and we invited a random sample of 200 participants from those who had agreed to do so (Table 1).

### Data collection

Introductory letters explaining the study were sent to members of the three samples for whom an address could be identified. Addresses were not available for those with mobile telephone numbers. The letter was followed by a telephone call by a trained interviewer inviting the potential participant to complete a computer-assisted telephone interview (CATI). Where no address was available the potential participant was called without a prior introductory letter. Up to ten phone call attempts to contact the potential participant were made at different times of day and days of the week. Participation was voluntary and interviews could be terminated at any time.

An initial questionnaire was drafted based on that used in a previous Canadian study [18] and it was modified following qualitative research that we conducted in order to identify questions of relevance to Australian participation in health research [19].

Our questionnaire commenced with an initial general question about willingness to participate in health research and there were 12 follow-up questions seeking to identify factors that affected willingness. These included the reason for the research e.g., treatment or prevention of disease or planning hospital services; whether the disease was one they or a close friend or relative had experienced; the funding source for the research e.g., charity, government or pharmaceutical industry; and how the individual had been selected to take part in the study e.g., randomly selected or from their General Practitioner (GP). Participants were also asked whether receipt of a summary of the research results would make them more willing to take part. All responses were scored from 1 to 5. One was Very Unwilling, two was Unwilling, three was Neutral, four was Willing and five was Very Willing to participate. Participants were then asked whether, if they were to take part in research, they would prefer shorter or longer explanatory statements about the research and whether they would prefer a telephone survey, postal survey or an online survey.

We asked for demographic details (age, sex, highest level of formal education, postal area code of residential address, Aboriginal and Torres Strait Islander (ATSI) status, and country of birth), whether they had a long term disease or disability and their previous participation in health research.

Postal area code data were used to assign the Socio-Economic Indexes for Areas (SEIFA), an aggregate index of relative socio-economic disadvantage [20] and to group individuals by remoteness [21]. National comparison data were drawn from Australian Bureau of Statistics (ABS) data released in 2008 for education [22] and in 2012 for sex, age and ATSI status [23, 24].

Analysis was carried out using Stata version 11.2 [25]. For questions about willingness to participate, Very Unwilling and Unwilling responses were grouped together, as were Very Willing and Willing. This resulted in a three level ordinal outcome variable of willingness to participate. Assessment of associations between candidate binary predictor variables and the ordered outcome variable was performed with chi-squared tests for trend, and ordinal logistic regression was used for the ordered predictor variables (age, remoteness and SEIFA). Assessment of other associations between non-ordered categorical variables was performed using conventional chi-squared tests.

A multivariable analysis of predictors of being willing or very willing to participate was performed using logistic regression for each participant group separately.

Ethics Committee approval was granted by Monash University Human Research Ethics Committee (HREC), by University of Western Australia Ethics Committee in respect of the Case-control study and by relevant Ethics Committees for the Aboriginal and Torres Strait Islander focus groups.

## Results

After excluding individuals who could not be contacted, there were a total of 4171 eligible participants of whom 2103 (50.4 %) participated. The participation fraction was 44.5 % for the general population sample and over 80 % for those who had participated in previous epidemiological studies. (Table 1) There was no significant difference in the participation fraction between people who we contacted using their mobile number and landline number (*p* = 0.85). For the general population sample with a landline, the response was 5 % greater in those for whom we had an address, and sent a letter, than for those for whom we did not have an address and did not send an introductory letter prior to contacting them.

Table 2 shows that in comparison with the Australian general population, our general population sample had a greater proportion of women than men (58 vs 51 %), were more likely to come from outer regional, remote or very remote areas (21 vs 12 %), had a higher level of tertiary education (35 vs 24 %) and were more likely to be in the most socio-economically advantaged quintile (25 vs 20 %). Three quarters of the general population sample had not previously participated in a health survey and a similar proportion did not have a long-term disease or disability. Only 1 % in each of the three study groups spontaneously raised questions about privacy.

**Table 2:** Demographic characteristics of participants

	Australian population % (ABS data) [20–24]	General population sample % *n* = 1559	Case-control study sample % *n* = 153	Cohort study sample % *n* = 401
	Aged ≥18			
Male^a^	49	42	61	94
Female	51	58	39	6
Age^a^ 18–39 years	40	28	0	1
40–60 years	34	41	13	39
>60 years	26	31	87	60
ATSI:^ab^ Yes	2.5	1	2	1
No	97.5	99	98	99
Country of birth^a^				
Australia	73	77	70	75
Other	27	23	30	25
Highest level of education^a^	25–64 years only			
School	41	42	49	46
Technical/trade certificate	35	22	30	32
Tertiary	24	35	21	22
Other	-	1	0	0
Remoteness index^a^				
Metropolitan	69	56	61	68
Inner regional	20	22	28	24
Outer regional/Remote	12	21	10	8
SEIFA Relative Disadvantage^ac^				
Quintile 1 least advantaged	20	14	2	10
Quintile 2	20	20	13	18
Quintile 3	20	22	27	18
Quintile 4	20	19	22	24
Quintile 5 most advantaged	20	25	36	30
Previously done health survey Yes		26	100	100
No		74	0	0
Previously refused health survey Yes		11	8	10
No refusal		89	92	90
Long-term disease/disability Yes		25	43	30
No		75	56	71

^a^Chi squared test *p* < 0.05 of comparison between the Australian population (column 2) and the general population sample (column3)

^b^Aboriginal or Torres Strait Islander

^c^SEIFA, Socio-Economic Indexes for Areas is an aggregate score of relative socio-economic disadvantage; the lower the score the higher the relative disadvantage

Most of the case-control study respondents were over 60 years of age and 60 % were male (Table 2). Consistent with the composition of the cohort, most (94 %) of the cohort respondents were male and the majority were over 60 years of age.

Nearly two-thirds (61 %) of the general population sample stated that they would be willing or very willing to participate in health surveys and only 11 % said that they were generally unwilling (Table 3). A higher proportion, 83 %, of the participants drawn from the cohort and case control study were willing to participate in another study.

**Table 3:** Stated willingness to participate in health studies by demographic characteristics

	General population row %					Case-control study participants row %					Cohort study participants row %				
	Number	Very unwilling/Unwilling	Neutral	Very willing/Willing	*p* value^a^	Number	Very unwilling/Unwilling	Neutral	Very willing/ Willing	*p* value^a^	Number	Very unwilling/Unwilling	Neutral	Very willing/Willing	*p* value^a^
Whole group	1546	11	28	61		153	1	5	93		401	13	69	319	
Male	653	12	30	58	0.047	93	2	2	96	0.182	375	3	65	297	0.469
Female	893	10	27	61		60	0	10	90		26	0	4	22	
Age <40 years	428	12	36	52	<0.001	0	0	0	0	0.992	3	0	67	33	0.056
40–60 years	637	11	29	60		20	0	0	100		156	3	21	76	
>60 years	475	10	20	70		132	2	6	92		241	3	14	83	
ATSI^b^: Yes	22	18	18	64	0.967	3	0	0	100	0.643	3	0	67	33	0.059
No	1512	11	28	61		148	1	5	93		393	3	17	80	
Country of birth															
Australia	1181	11	27	61	0.495	107	1	7	93	0.511	285	3	18	79	0.483
Other	359	11	31	59		45	2	2	96		116	3	15	82	
Highest level education															
School	650	13	26	61	0.218	74	2.7	7	91	0.356	183	2	16	82	0.400
Technical/trade certificate	341	9	35	57		46	0	2	98		126	6	17	77	
Tertiary	535	10	27	64		31	0	6	94		89	2	19	79	
Other	10	10	20	70		0	0	0	0		0	0	0	0	
Remoteness index															
Metropolitan	870	11	30	59	0.291	94	1	6	93	0.373	272	4	17	79	0.680
Inner regional	345	12	24	64		43	2	5	93		98	3	18	79	
Outer regional/Remote	331	11	28	62		16	0	0	100		31	0	16	84	
SEIFA Relative Disadvantage^c^															
Quintile 1 most disadvantaged	217	15	29	57	0.287	3	0	0	100	0.928	39	5	23	72	0.618
Quintile 2	312	9	29	62		20	0	5	95		73	1	8	90	
Quintile 3	340	11	28	61		42	2	5	93		72	0	22	78	
Quintile 4	293	10	31	59		33	3	6	91		97	6	19	75	
Quintile 5 least disadvantaged	384	12	25	64		55	0	5	95		120	3	17	80	
Previously done health survey Yes	384	6	21	73	<0.001	153	1	5	94	-	401	3	17	80	-
No	1107	13	30	57		0	0	0	0		0	0	0	0	
Previously refused health survey Yes	166	19	33	48	<0.001	11	0	9	91	0.585	38	5	18	76	0.501
No refusal	1329	10	27	63		135	0	5	95		355	3	17	81	
Long-term disease/disability Yes	481	9	23	68	0.002	65	2	2	97	0.131	115	4	16	80	0.967
No	1150	12	30	59		85	1	8	91		278	3	18	80	
Spontaneously raised privacy/health issue during interview Yes	19	26	21	53	0.245	2	0	0	100	0.708	5	20	40	40	0.021
No	1527	11	29	61		151	1	5	93		396	3	17	80	

^a^A chi squared test for trend was used for comparing the numbers for all variables except age, Remoteness index and SEIFA for which ordinal logistic regression was used

^b^ *ATSI* is the acronym for Aboriginal and Torres Strait Islander

^c^ *SEIFA* Socio-Economic Indexes for Areas is an aggregate score of relative socio-economic disadvantage; the lower the score the higher the relative disadvantage

For the general population sample, greater willingness to participate was reported by women than men (61 vs 58 % *p* = 0.047), older than younger people, and those with a long term disease or disability. There was no significant difference in initial willingness to participate by country of birth, level of education or remoteness (Table 3). In the general population sample, the lowest stated willingness by age and sex was 46 %, for men under the age of 40, the highest was 72 %, for men over 60. Previous participation or not having previously refused to participate in a health survey was also a strong predictor of willingness to participate.

Similar results in terms of willingness to participate by most demographic characteristics were seen for the case-control and cohort participants (Table 3). For both groups of previous health survey participants however, long term disease or disability did not increase their willingness to participate. The previous cohort participants who were unwilling to participate were more likely to raise privacy spontaneously during the interview (*p* = 0.021) although numbers were small (1.3 %).

The multivariable analyses revealed very little difference in the importance of any predictor. Exceptions were for the general population group where long term disability was no longer significant due to adjustment for age (*p* = 0.13), and for the cohort population raising privacy issues which did not retain its statistical significance (*p* = 0.06). In the case control study there were too few participants reporting lack of willingness to participate in order to perform a reliable multivariable analysis.

**Fig. 1:**
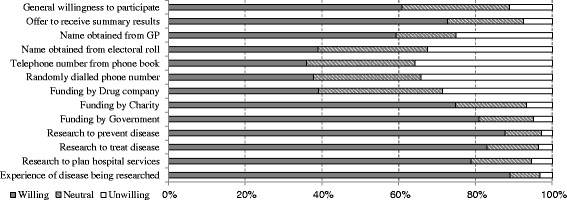
Percent of the general population participants who were willing to take part in health research, by whether they would receive a summary of results, by how their name/phone number had been identified (*4 options*), by method of study funding (*3 options*), by reason for the research (*3 options*) and by whether the person had direct experience/knowledge of the disease being investigated

Factors which increased participants’ stated willingness to participate in health surveys (Fig. 1) were: research to treat or prevent a disease or to better plan hospital services; research about a disease they or a close family member or friend had experienced; government or charity funded research; receipt of a summary of the results.

With respect to mode of contact, people were more willing to have their name provided to researchers by their GP when diagnosed with a particular disease, than to have their name taken from the electoral roll, the telephone directory or have their telephone number randomly generated.

The majority of participants in each age and sex group expressed preference for an in-depth 4-page over a brief 1-page study explanation (Table 4). Both men and women, particularly younger people, expressed a preference for an online survey over a telephone survey. Older people, particularly women, preferred a mail survey. A telephone survey was the least preferred method for all subgroups except those over 60 years who least favoured an online survey.

**Table 4:** Australian general population sample preference for study explanation, and survey method by sex and age group ( % by column)

	Total	Men	Women	<40 years	40–60 years	>60 years
	*n* (%)	*n* (%)	*n* (%)	*n* (%)	*n* (%)	*n* (%)
Study explanation						
1-page explanation	631 (42)	276 (43)	355 (40)	182 (43)	244 (39)	200 (43)
4-page explanation	838 (55)	339 (53)	499 (57)	230 (54)	367 (59)	240 (52)
Don’t know/care	51 (3)	24 (4)	27 (3)	11 (3)	15 (2)	25 (5)
		*P* ^a^= 0.343		*P* ^a^= 0.018		
Survey method						
Telephone survey	310 (20)	140 (22)	170 (19)	70 (16)	118 (19)	122 (26)
Mail survey	498 (32)	182 (28)	316 (35)	79 (18)	193 (30)	225 (47)
Online survey	623 (40)	284 (44)	339 (38)	256 (60)	277 (44)	87 (18)
Don’t know/care	111 (7)	43 (7)	68 (8)	23 (5)	48 (8)	40 (8)
		*P* ^a^= 0.011		*P* ^a^<0.001		

^a^Chi squared

## Discussion

Overall, the majority (61 %) of participants in our study were willing to participate in health research and only about 11 % said that they were unwilling.

Participation in our study was higher for those who had previously participated in health research across two methodologies, a case-control or cohort health study, than for the general population sample. This high rate of participation by people recruited from our previous studies suggests that their participation in a health study had not diminished their willingness to participate. In the case of the largely male cohort, the responders continued to be willing to take part in research, although men usually show lower interest in participating in health research than women*,* as seen among the general population participants in this study. Case-control participants had a higher participation rate in this survey than the cohort participants which may be a result of the shorter time between the case-control study and our survey. It may also reflect the reduced interest in participating in a study which is less personally relevant than the cohort study. There may be value in ensuring that all study participants are canvassed at set up as to their willingness to participant in future studies, as was done in the WABoHS study, and these individuals may provide a pool for future study recruitment.

A greater proportion of the case-control study participants (43 %) had a long term disease or disability than did the general population sample (25 %) or the cohort study population (30 %) but an individual’s case-control status was unknown in this study. Those who were cases may have been motivated by their experience of a serious disease.

Some questions such as education level or long term disability may have been subject to reporting bias but the remoteness index and SEIFA were coded from area measures and are not likely to be biased.

A limitation of our study was the 44.5 % participation rate for the general population sample. Those who refused to take part in our study may also be unwilling to take part in other health research and/or might have taken part if it had not been a telephone survey. Response rates may have decreased in bona fide health research because of confusion with increased telemarketing ‘surveys’ or because of reduced volunteering more generally in society [4]. An investigation of public opinion in Canada published in 2010 included 3000 participants selected from the telephone directory with 58.3 % participation of those eligible, while Molster et al. (2007) [18] obtained a 78 % response rate from 600 participants in a study of the views of Western Australians on the collection of identifiable data [26].

This form of research clearly involves some circularity, in that those who took part are de facto, willing to participate. However, it is difficult to see how we can explore people’s views without asking them and there were informative differences in rates of willingness associated with different questions and different subgroups of participants. Interviewees were advised about the overall aims of the study before giving consent to participate and it may be that the particular topic might have induced them to participate when ordinarily they would not have agreed. Researchers have suggested that factual willingness to participate in health research is greater than hypothetical willingness. That is, a higher proportion of people actually take part in research when asked than say they are willing do so in answer to a hypothetical question [27]. In support of this, 11 % of the general population sample who took part in our survey said they were unwilling or very unwilling to take part in health research.

We have no information about the individuals who did not answer the telephone or who answered but did not take part in the study. The telephone numbers were obtained from a commercial list without such information as age or sex. In view of this we cannot assess any non-response bias. We have compared the demographic descriptors of our general population sample to those of the general population to assess how different they are to the general population however. Our general population survey respondents were more likely to be female and older than the Australian population. Participation differed with measures of socio-economic disadvantage and by individual level educational attainment. These demographic differences in participation have been found in other studies [12, 28–32].

We found a very modest increase in response when personalised letters were sent before the telephone survey to those for whom an address was available. We did not randomise for those with and without an address and these groups may differ in regards to willingness to respond. Randomisation would have enabled us to assess whether receiving a letter enhanced participation in our study. This is consistent with a systematic review [33] and subsequent study [34] which found that personalised questionnaires and letters increased the response for postal questionnaires. However, a recent randomised controlled trial in Australia, showed that letters did not increase the participation rate in an epidemiological study using telephone interviews [35].

People were most likely express willingness to participate if the research was about treating or preventing a disease, or if it was about a disease that they or a family member or friend had experienced. Expressed willingness was increased when the question provided more information about the reason for the study, (hospital planning, treatment or prevention of a disease) or about the source of the funding, (government or charity) or if the participants were to receive a copy of the results. These findings seem to indicate that people are more prepared to take part in studies where the reason for the research is evident and the findings will be made available.

Australians in our study were less likely to be willing to participate in research sponsored by a pharmaceutical company than by a university or charity. Willison et al., in a survey of Canadian general practice patients, also found concern about their records being used for research by drug companies [36]. Canadians were also less willing to take part in research sponsored by other private sources such as a private research firm [18] or a private insurance company [36]. In a systematic review of randomised controlled trials of postal questionnaires, those originating from universities were about 30 % more likely to be completed and returned than were questionnaires from other sources [33].

Many of our participants, like those surveyed in Canada [18], wanted a copy of the summary results from the research. Kaufman et al. [37] found reduced willingness to participate if respondents were told that they would not receive their individual research results and their reduced willingness was related to privacy concerns. Very few of our participants raised concerns about privacy during the interview.

Difficulty in identifying and contacting potential participants is a barrier to participation. We used randomly selected telephone numbers for all the general population participants, but fewer than 40 % of these participants indicated that this would be a contact methodology that would make them favour participation (Fig. 1).

Our study asked participants about their preferences for survey delivery, i.e., online or mail and we found that younger participants expressed a preference for online surveys but this has not always been successful strategy for recruitment in practice [4]. A recent web-based survey of mental health among Australian University students had a 25 % participation rate [38]. Newer survey methods such as web based questionnaires have advantages and disadvantages in comparison with traditional survey methods such as telephone or postal questionnaires but have been used considerably less commonly in comparison and there are concerns about nonresponse bias and reliability of the data collected by this method [39]. Our study has provided contemporary information using an established method to inform future recruitment to health research studies.

Finally, the preference in every group for an in-depth 4-page over a brief 1-page study explanation came as a surprise, given the concern often expressed over the lengthy, potentially confusing information that is often required to be provided to participants. If the contrast had been between 1, 4, and 12 pages, the implications of this finding might have been clearer.

Reduced participation over time has been encountered across all epidemiological study designs, in terms of non-response as well as refusal, and a low response rate may reduce the statistical power and generalisability of results to the wider community. Therefore it is important to maintain current understanding of people’s willingness to participate in health research and factors that affect their willingness to participate.

Despite declining participation rates, the fact that participation in health research studies is seen positively is a good sign. Our findings have several implications for future research with a view to improving participation in health research studies. We suggest that the bone fide credentials of health researchers or their institutions are identified early in the interview process to distinguish them from telemarketing ‘surveys’. Further that the reason for the call is communicated clearly and is understandable to participants. The source of funding should be identified, and if the funding source is not a profit-making entity this should be emphasised. Participants should be made aware of the ways that the findings will be made available. Participants should be offered a range of methods of data collection (where feasible) eg offering the options of telephone, postal and web based survey. Different demographic groups may need to be targeted with different recruitment methodologies. A one page summary plain language statement should be provided alongside a longer (4–5 page) explanation for those who wished to have more information. Some individuals may have been motivated to participate by their knowledge of a serious disease or disability. Information materials should adequately describe, in lay terms the nature of the disease and the implications for sufferers of the disease. This may be particularly important for controls. Finally, researchers should ask participants at the time of enrolment to studies to agree to be contacted for future surveys.

## Conclusions

The majority of respondents to this survey indicated that they were willing or very willing to participate in health research. The experience of previous participation in epidemiological surveys did not reduce willingness to participate, indeed there was a much higher response rate among previous participants in a cohort and a case control study.

Participation rates in telephone surveys have fallen over time. Our survey only included those willing to take part in a survey, but it is encouraging that those who have already taken part in health research are highly responsive and very willing to take part in another study. Our study could also inform practice to improve participation in epidemiological health research and participants’ experience of their participation.

## Abbreviations

ABS: Australian Bureau of Statistics
ATSI: Aboriginal and Torres Strait Islander
CATI: Computer-assisted telephone interview
GP: General practitioner
SEIFA: Socio-economic indexes for areas

## References

[1] Morton LM, Cahill J, Hartge P (2005). Reporting participation in epidemiologic studies: A survey of practice. Am J Epidemiol.

[2] Jones J (1996). The effects of non-response on statistical inference. J Health Soc Policy.

[3] Kjoller M, Thoning H (2005). Characteristics of nonresponse in the Danish Health Interview Surveys, 1987–1994. Eur J Public Health.

[4] Galea S, Tracey M (2007). Participation rates in epidemiologic studies. Ann Epidemol.

[5] O’Toole J, Sinclair M, Leder K (2008). Maximising response rates in household telephone surveys. MBMC Med Res Methodology.

[6] Boyle T, Landrigan J, Bulsara C, Fritschi L, Heyworth J (2011). Increasing study participation. Epidemiology.

[7] Curtin R, Presser S, Singer E (2005). Changes in telephone survey nonresponse over the past quarter century. Pub Op Quart.

[8] Harris MA, Levy AR, Teschke KE (2008). Personal privacy and public health: potential impacts of privacy legislation on health research in Canada. Can J Pub Health.

[9] Madigan MP, Troisi R, Potischman N, Brogan D, Gammon MD, Malone KE (2000). Characteristics of respondents and non-respondents from a case–control study of breast cancer in younger women. Int J Epidemiol.

[10] Kho ME, Duffett M, Willison DJ, Cook DJ, Brouwers MC (2009). Written informed consent and selection bias in observational studies using medical records: systematic review. BMJ.

[11] Kypri K, Samaranayaka A, Connor J, Langley J, Maclennan B (2011). Non-response bias in a web-based health behaviour survey of New Zealand tertiary students. Prev Med.

[12] Maclennan B, Kypri K, Langley J, Room R (2012). Non-response bias in a community survey of drinking, alcohol-related experiences and public opinion on alcohol policy. Drug Alcohol Depend.

[13] Fritschi L, Kelsall H, Loff B, Slegers C, Zion D, Glass D (2015). A cross-sectional survey to investigate community understanding of medical research ethics committees. J Med Ethics.

[14] Glass DC, Gray CN, Jolley DJ, Gibbons C, Sim MR, Fritschi L (2003). Leukemia risk associated with low level benzene exposure. Epidemiology.

[15] Glass DC, Wood E, Del Monaco A, Sim M. Cohort Profile: Health Watch-a 30-year prospective cohort study of Australian 5 petroleum industry workers. Int J Epidem. 2015. 10.1093/ije/dyv121 26157111

[16] Iacopetta B, Heyworth J, Girschik J, Grieu F, Clayforth C, Fritschi L (2009). The MTHFR C677T and ΔDNMT3B C-149 T polymorphisms confer different risks for right- and left-sided colorectal cancer. Int J Cancer.

[17] Boyle T, Fritschi L, Platell C, Heyworth J (2013). Lifestyle factors associated with survival after colorectal cancer diagnosis. Br J Cancer [Epidemiology].

[18] Teschke K, Marino S, Chu R, Tsui J, Harris MA, Marion S (2010). Public opinions about participating in health research. Can J Pub Health.

[19] Slegers C, Zion D, Glass D, Kelsall H, Fritschi L, Brown N (2015). Why do people participate in epidemiological research?. J Bioethical Inquiry.

[20] Australian Bureau of Statistics (2012). 2039.0 - Information Paper: An Introduction to Socio-Economic Indexes for Areas (SEIFA), 2006.

[21] Australian Bureau of Statistics (2012). 1216.0 - Australian Standard Geographical Classification (ASGC) - Electronic Publication, 2005.

[22] Australian Bureau of Statistics (2008). 4102.0 Australian Social Trends, Data Cube.

[23] Australian Bureau of Statistics (2012). 3105.0.65.001 Australian Historical Population Statistics, September 2011.

[24] Australian Bureau of Statistics (2012). 4102.0 Australian Social Trends, Data Cube - Population 1998–2011.

[25] StataCorp (2011). Stata Statistical Software: Release 11.2. Version.

[26] Molster C, Bower C, O’Leary P (2007). Community attitudes to the collection and use of identifiable data for health research – is it an invasion of privacy?. Aust NZ J Pub Health.

[27] Johnsson L, Helgesson G, Rafnar T, Halldorsdottir I, Chia K-S, Eriksson S (2010). Hypothetical and factual willingness to participate in biobank research. Eur J Hum Genetics.

[28] Angus V, Entwistle V, Emslie M, Walker K, Andrew J (2003). The requirement for prior consent to participate on survey response rates: A population-based survey in Grampian. BMC Health Serv Res.

[29] Sogaard A, Selmer R, Bjertness E, Thelle D (2004). The Oslo Health Study: The impact of self-selection in a large, population-based survey. Int J Equity Health.

[30] van der Waerden JEB, Hoefnagels C, Jansen MWJ, Hosman CMH (2010). Exploring recruitment, willingness to participate, and retention of low-SES women in stress and depression prevention. BMC Public Health.

[31] Shickle D, Carlisle J, Wallace S, Cork M, Beyleveld D, Bowns I (2002). Patient Electronic Record: Information and Consent (PERIC) Public attitudes to protection and use of personal health information.

[32] Iversen A, Liddell K, Fear N, Hotopf M, Wessely S (2006). Consent, confidentiality, and the Data Protection Act. BMJ.

[33] Edwards P, Roberts I, Clarke M, DiGuiseppi C, Pratap S, Wentz R (2007). Methods to increase response rates to postal questionnaires.[see comment]. Cochrane Database Syst Rev.

[34] Edwards S, Slattery M, Edwards A, Sweeney C, Murtaugh M, Palmer L (2009). Factors associated with response to a follow-up postal questionnaire in a cohort of American Indians. Prev Med.

[35] Carey R, Glass DC, Reid A, Benke G, Fritschi L (2013). An advance letter did not increase response rates in a telephone survey: A randomised trial. J Clin Epidemiol.

[36] Willison DJ, Keshavjee K, Nair K, Goldsmith C, Anne M (2003). Holbrook for the COMPETE investigators. Patient consent preferences for research uses of information in electronic medical records: interview and survey data. BMJ.

[37] Kaufman DJ, Murphy-Bollinger J, Scott J, Hudson KL (2009). Public opinion about the importance of privacy in biobank research. Am J Hum Genetics.

[38] Said D, Kypri K, Bowman J (2013). Risk factors for mental disorder among university students in Australia: findings from a web-based cross-sectional survey. Soc Psychiatry Psychiatr Epidemiol.

[39] van Gelder MMHJ, Bretveld RW, Roeleveld N (2011). Abstracts of the 3rd North American Congress of Epidemiology, June 21–24, 2011 Montreal, Canada Web-based Questionnaires: The Future in Epidemiology?. Am J Epidemiol.

